# The Importance of Next-Generation Sequencing in Identifying Immunohistochemically Ambiguous Pediatric Sarcomas

**DOI:** 10.1155/crom/9926653

**Published:** 2025-03-13

**Authors:** Chong Bin He, Dan Pham, Rachel S. Kronenfeld, Andrew Rosenberg, Jessica Ardente, Aditi Dhir

**Affiliations:** ^1^Department of Pediatrics, University of Miami Miller School of Medicine/Jackson Memorial Hospital, Miami, Florida, USA; ^2^Division of Pediatric Hematology/Oncology/Bone Marrow Transplantation, University of Miami Miller School of Medicine/Jackson Memorial Hospital, Miami, Florida, USA; ^3^Division of Pediatric Pathology, University of Miami Miller School of Medicine/Jackson Memorial Hospital, Miami, Florida, USA

**Keywords:** cancer diagnosis, cancer pathology, clear cell sarcoma, clinical pathology, Ewing sarcoma, immunohistochemistry, next-generation sequencing, pediatric sarcoma, soft-tissue sarcoma

## Abstract

Bone and soft-tissue sarcomas encompass over 70 histologic subtypes, posing diagnostic challenges due to overlapping characteristics. Molecular analyses, such as fluorescence in situ hybridization (FISH) and reverse transcription polymerase chain reaction (RT-PCR), aid in identifying specific genomic alterations but are often limited, particularly when prior histological findings are inconclusive. Next-generation sequencing (NGS) offers high-throughput testing via a targeted sequencing panel, addressing these limitations. This case series highlights the utility of NGS in diagnosing two pediatric patients with immunobiologically ambiguous Ewing sarcoma (ES) and clear cell sarcoma (CCS), emphasizing its role as a powerful tool in solid tumor diagnosis.

## 1. Introduction

Sarcomas are a heterogeneous group of mesenchymal tumors that arise in bone and soft tissues, including connective tissue, muscle, nerves, blood vessels, and fat. They account for 13%–20% of all pediatric solid malignancies. Many soft tissue and bone sarcomas are aggressive, contributing to cancer-related mortality and morbidity. While the most common pediatric sarcomas are rhabdomyosarcoma, osteosarcoma, and Ewing sarcoma (ES), more than 70 histologic subtypes have been described. Diagnosing sarcomas can be challenging due to overlapping clinical, histological, and immunohistochemical characteristics, which complicate differentiation among subtypes.

Molecular analyses such as reverse transcription polymerase chain reaction (RT-PCR); fluorescence in situ hybridization (FISH); and, most recently, next-generation sequencing (NGS) have played a crucial role in improving the diagnostic accuracy of sarcoma [1–3]. In recent years, NGS has been increasingly utilized in cancer research and hospital settings due to improved turnaround times and reduced costs [3, 4]. In the field of bone and soft tissue sarcoma, NGS enables the rapid identification of multiple molecular alterations, including disease-specific translocations, amplifications, and mutations within a silent genomic background. Emerging cases in the literature illustrate the utility of NGS in identifying solid tumors that have perplexing morphologic and immunophenotypic features [5, 6].

Despite its powerful diagnostic potential, NGS remains underutilized in clinical settings, particularly for solid tumor diagnosis, as reflected in the limited cases reported in the literature. In this case series, we highlight the successful use of NGS to accurately diagnose two pediatric patients with histologically and immunobiologically ambiguous soft tissue masses, ultimately guiding disease-specific therapeutic management.

## 2. Case Presentations

### 2.1. Patient 1

**Figure 1:**
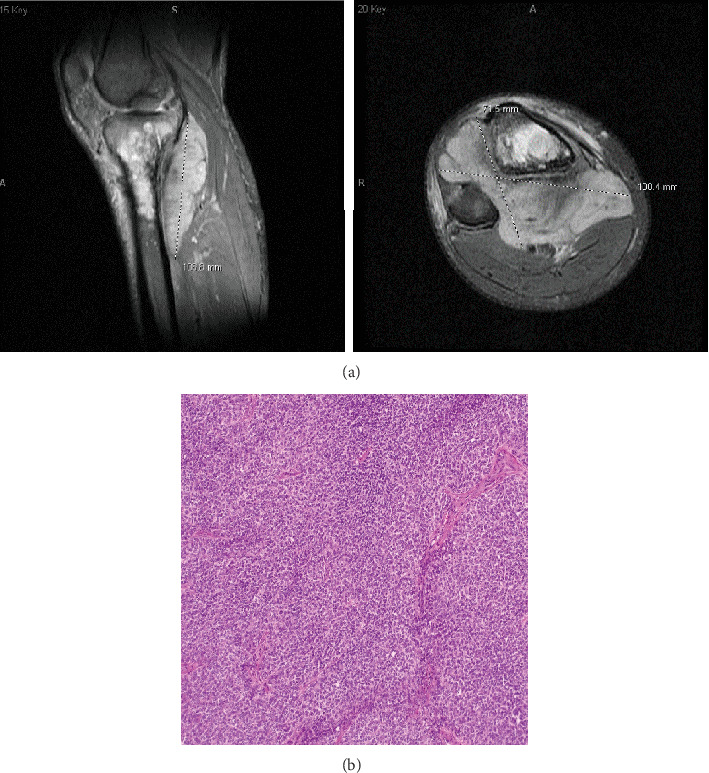
(a) MRI of the right tibia of Patient 1 at initial diagnosis. The right tibial mass measured 10.7 × 10.0 × 7.2 cm. (b) Hematoxylin and eosin staining of the right tibial mass of Patient 1 shows sheets of small round cells with round to oval nuclei containing fine chromatin and small amounts of eosinophilic cytoplasm.

A 16-year-old male with no significant past medical history presented with a 6-month history of right lower extremity pain and swelling. His x-ray showed mixed sclerotic and lytic lesions of the proximal right tibia. Magnetic resonance imaging (MRI) revealed a large lobulated mass within the proximal right tibia extending into the adjacent right tibialis anterior and posterior musculature measuring 10.7 × 10.0 × 7.2 cm (Figure 1(a)). Computed tomography (CT) of the chest showed no pulmonary involvement. However, a positron emission tomography (PET) scan demonstrated extensive metastasis to the adjacent musculature and distant lymph nodes in the pelvis. Biopsy showed poorly differentiated small round cell sarcoma that was positive for BCL-6 corepressor (BCOR), CD99, friend leukemia virus integration site 1 (FLI1), and transducin-like enhancer of split 1 (TLE1) (Figure 1(b)). Cells were negative for NKX2.2, suggestive of *BCOR::CCNB3* Ewing-like sarcoma (ELS). However, RNA-based NGS results, obtained via a CLIA-certified laboratory (Caris Life Sciences; Phoenix, Arizona) revealed the presence of a characteristic fusion between *EWS RNA-binding protein 1* (*EWSR1*) and *FLI1*, confirming the diagnosis of ES. The patient underwent eight cycles of chemotherapy per Children's Oncology Group AEWS1221 protocol with 55.8-Gy proton beam therapy in 31 fractions as definite local control, as he was not a surgical candidate. End-of-therapy imaging showed no active disease. However, most recent scans, 28 months posttherapy, revealed distant metastasis for which the patient is currently undergoing therapy.

### 2.2. Patient 2

**Figure 2:**
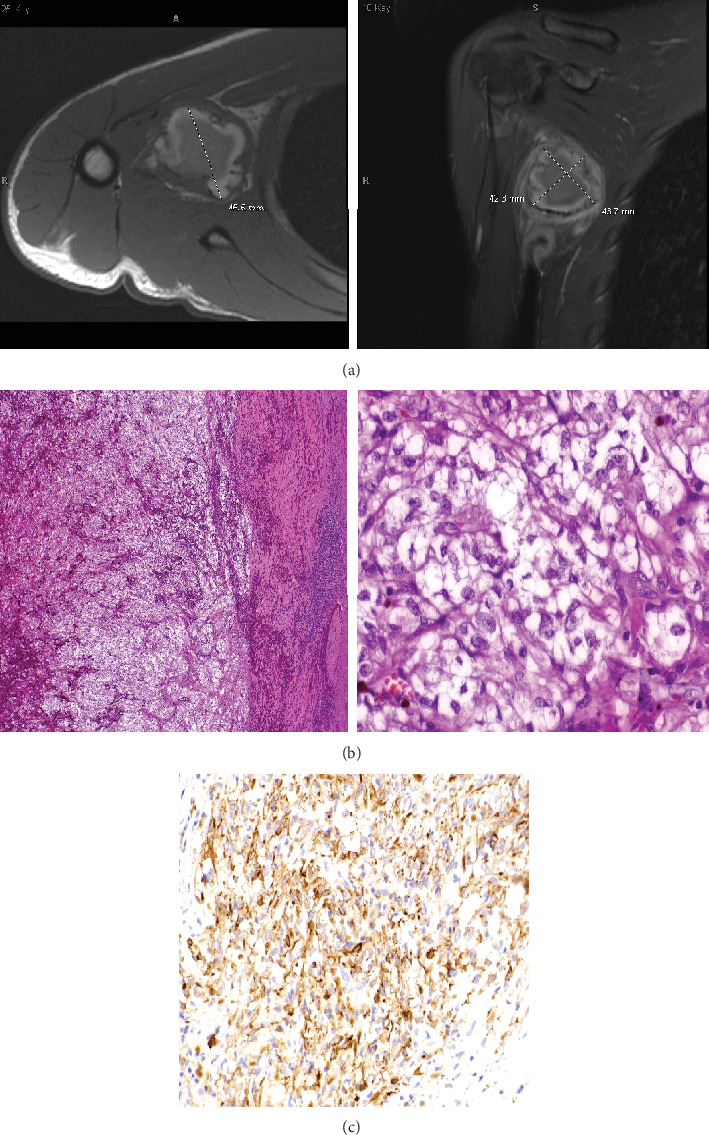
(a) MRI of the right axilla of Patient 2 at initial diagnosis. The right axillary mass measured 4.6 × 4.2 × 4.4 cm. (b) Hematoxylin and eosin staining of the right axillary mass of Patient 2 shows cells with abundant clear cytoplasm and oval to round nuclei containing fine chromatin. (c) Immunohistochemistry staining of the right axillary mass of Patient 2 shows cells positive for pankeratin and S100 protein.

An 18-year-old male with no past medical history experienced a four-year history of waxing and waning pain in the right arm and right hand and a 1-year history of a right axillary mass. MRI showed a round right axillary mass measuring 4.6 × 4.2 × 4.4 cm with peritumoral edema and enhancement but no central enhancement (Figure 2(a)). CT angiogram of the right upper extremity demonstrated a mass distinct from the right axillary artery which abutted the axillary, pectoral, and lateral thoracic vertebral branches without encapsulation. CT chest showed no pulmonary involvement. The patient underwent surgical resection of the right axillary tumor with a small portion (5%–10%) of the tumor deemed unresectable because it adhered to the brachial plexus. Histological evaluation demonstrated a well-circumscribed mass with central cystic hemorrhagic change and marked chronic inflammation with abundant hemosiderin, hematoidin, and sclerosis. The neoplastic cells were large, polyhedral, and arranged in sheets and groups and had abundant clear cytoplasm (Figure 2(b)). Cells were positive for pankeratin, keratin AE1/AE3, SOX10, and S100 and negative for HMB45 and MelanA (Figure 2(c)). FISH detected *EWSR1*-associated rearrangement, suggestive of either a clear cell myoepithelioma or clear cell sarcoma (CCS). Yet, the morphological features and immunohistochemical results were atypical for both possibilities. RNA-based NGS, conducted by CLIA-certified laboratory (Caris Life Sciences; Phoenix, Arizona), revealed the presence of the fusion of the *EWSR1* (22q12.1) and *ATF1* (12q13.12) loci, confirming the diagnosis of CCS. The patient, therefore, underwent a second surgery for complete surgical resection of the tumor mass and lymph node dissection of the surrounding axillary nodes. The margins were negative, and no lymph node involvement was observed. Follow-up MRI after the second surgery showed no recurrent lesion in the previously resected area. Adjuvant therapy was not recommended given the patient's complete resection and absence of advanced disease. The patient is undergoing disease surveillance every 3 months with no evidence of disease for about a year since resection.

## 3. Discussion

The first case represents a diagnosis of histologically ambiguous ES, initially misidentified as ELS before NGS confirmed the characteristic ES fusion. ES and ELS are highly aggressive mesenchymal neoplasms that primarily affect children and young adults [7]. In 2020, the World Health Organization newly classified both ES and ELS as undifferentiated small round cell sarcomas [8]. Due to the histological similarities, advanced diagnostic tools such as immunostaining and molecular profiling are essential for accurately differentiating these subtypes.

In children, ES is the second most common primary malignant bone tumor, predominantly affecting the metaphysis of the long bones, followed by the pelvis, ribs, and vertebrae [9]. It is characterized by the fusion of the *EWSR1* gene with other fusion partners, most commonly the *FLI1* gene. ES cells are monomorphic; round; highly mitotic; and strongly CD 99+, NXK2.2+, and TLE1−. Current treatment consists of compressed chemotherapy combined with local control through surgery and/or radiation [10]. In contrast, ELS lacks the characteristic *EWSR1* gene fusion found in ES, though other fusions and molecular alterations have been identified in different ELS subtypes. One such subtype, BCOR-CCNB3 sarcoma (BCS), commonly arises in the pelvis, lower limbs, and paraspinal region. Histologically, BCS contains a mix of round and spindle cells and is CCNB3+, BCOR+, and weakly CD99+. *BCOR::CCNB3* rearrangements account for 60% of BCOR gene alterations in BCS. Compared to ES, BCS follows a more indolent clinical course [7, 8, 11, 12].

The overlapping histological and clinical features of BCS and ES can make diagnosis challenging. In a study by Puls et al., 70% of BCS cases exhibited small primitive cell morphology similar to ES, and 60% were CD99+ on immunohistochemistry [13]. In our patient's case, the tumor consisted of small round cells and was CD99+, BCOR+, FLI1+, TLE1−, and NKX2.2−. While CD99 and FLI1 are common immunohistochemical markers for ES, they lack specificity. NKX2.2, a downstream target of the *EWSR1::FL1* fusion gene, has emerged as a highly sensitive immunohistochemical marker for ES. Russell-Goldman et al. reported that NKX2.2 is 100% sensitive for ES and helps distinguish it from synovial sarcoma due to its TLE1 negativity [10]. Given the immunohistochemistry results, without the assistance of NGS, our patient would have been mistakenly diagnosed with BCS, which albeit currently treated with ES-based protocols has a more favorable prognosis overall [7, 11].

The second patient case demonstrates the role of NGS in confirming an ambiguous diagnosis of CCS, a soft tissue sarcoma that predominantly affects adults, with a median age of 39 years. Adolescents and young adults are rarely affected [14, 15]. CCS most commonly arises in the lower extremities but can also involve the head, neck, upper extremities, chest wall, and internal organs in rare cases. Grossly, CCS spears as a firm tan–gray mass. Histologically, CCS originates from neural crest cells and shares morphological features with malignant melanoma. Both tumors contain polygonal and fusiform cells with clear cytoplasm due to glycogen accumulation, as well as a centrally located round nucleus. CCS cells typically express HMB45 and S100 and may also be positive for CD99, MelanA, neurospecific enolase, and vimentin, while generally negative for cytokeratin, epithelial membrane antigen, carcinoembryonic antigen, desmin, and smooth muscle actin. Most CCS cases harbor the t(12;22)(q13;q12) translocation, resulting in the *EWSR1::ATF1* gene fusion, which serves as a key distinguishing feature from malignant melanoma [2, 15]. Several aspects of our patient's case were atypical for CCS, including the patient's young age (18 years), tumor location (right upper extremity), histological feature (mass with central cystic hemorrhagic change), and immunohistochemistry profile (pankeratin+, AE1/AE3+, S100+, HMB45−, MelanA−). The characteristic *EWSR1::ATF1* gene fusion identified by NGS resolved the diagnostic ambiguity, allowing for a definitive diagnosis and timely treatment. This is crucial, as CCS is highly infiltrative and has a high metastatic potential.

NGS is increasingly regarded as a “molecular microscope” for sarcoma diagnosis, as many sarcomas exhibit heterogeneous morphology and ambiguous immunohistochemistry profiles [4]. Advances in molecular biology have propelled cancer research forward. In an observational study by Italiano et al., histological diagnoses made by a sarcoma-expert pathologist were compared with diagnoses based on genomic hybridization, FISH, and RT-PCR. Of the 384 sarcoma cases, molecular testing altered 53 diagnoses and treatment plans [16]. Mathias et al. reported a poorly differentiated round cell sarcoma with an *EWSR1* gene rearrangement on FISH and CD99 positivity on immunohistochemistry, initially suggesting an ELS. However, NGS revealed a complex genome lacking the characteristic *EWSR1*-associated rearrangements and instead identified deletions in *RB1*, *PTCH1*, and *ATRX*, confirming the diagnosis of osteosarcoma [6]. In another case, Doyle et al. used NGS to reclassify an atypical lung mass initially diagnosed as an atypical carcinoid tumor. The detection of an *EWSR1::ERG* chromosome translocation led to the revised diagnosis of ES, an exceedingly rare occurrence in the lung [5].

NGS has revolutionized the biomedical field by enabling high-throughput sequencing of DNA and RNA fragments. In clinical settings, targeted NGS panels are more commonly used than whole-genome sequencing. NGS surpasses traditional molecular tests such as FISH and RT-PCR, which analyze only one gene per reaction. In contrast, NGS interrogates multiple genes simultaneously, reducing the amount of tissue and genetic material required. For tumors with multiple mutations or uncertain pathology, testing every possible alteration with FISH and RT-PCR is impractical. NGS, however, can perform targeted sequencing of dozens or hundreds of genes, significantly enhancing diagnostic accuracy. Furthermore, transcriptome-level RNA-based NGS can detect gene fusions and rare genomic aberrations that FISH, RT-PCR, and DNA-based NGS cannot. For example, as demonstrated in our patient case and others, NGS analysis with fusion-specific probes can distinguish ES from ELS in specimens with round cell sarcoma [3, 17, 18].

Despite its immense diagnostic utility, NGS remains costly and is often unavailable for on-site testing. Additionally, its turnaround time is longer (typically weeks) compared to microscopic evaluation, immunohistochemistry, and other molecular studies [17]. As more cancer-related genes and translocations are identified, the NGS diagnostic platform will become increasingly accurate, efficient, and cost-effective by expanding targeted sequencing panels. Beyond its diagnostic capabilities, NGS is paving the way for precision oncology, facilitating patient-specific targeted therapies based on tumor molecular profiles [18]. Other applications include identifying molecular mechanisms of treatment resistance and recurrent mutations driving tumorigenesis, which can greatly influence the management of recurrent cancers [4].

## 4. Conclusion

In this case series, we describe two uncommon clinical presentations of ES and CCS that could have been misdiagnosed and potentially mismanaged without NGS analysis. Establishing a precise diagnosis has important prognostic and therapeutic implications. This case series underscores the role of NGS in accurately diagnosing histologically and immunohistochemically ambiguous soft tissue masses. Pediatric oncology providers should be encouraged to incorporate NGS into the initial diagnostic evaluation for soft tissue masses, particularly when clinical, histological, and immunohistochemical features do not align with a definitive diagnosis.

## Data Availability

The authors have nothing to report.
